# Development and validation of a purification system for functional full-length human SR-B1 and CD36

**DOI:** 10.1016/j.jbc.2023.105187

**Published:** 2023-08-23

**Authors:** Hayley R. Powers, Shawn E. Jenjak, Brian F. Volkman, Daisy Sahoo

**Affiliations:** 1 Department of Biochemistry, Medical College of Wisconsin, Milwaukee, Wisconsin, USA; 2 Division of Endocrinology & Molecular Medicine, Department of Medicine, Medical College of Wisconsin, Milwaukee, Wisconsin, USA; 3 Cardiovascular Center, Medical College of Wisconsin, Milwaukee, Wisconsin, USA

**Keywords:** scavenger receptor, lipoprotein receptor, protein purification, atherosclerosis, glycosylation

## Abstract

Scavenger receptor class B type 1 (SR-B1) and CD36 are both members of the class B scavenger receptor family that play important roles in lipoprotein metabolism and atherosclerotic disease. SR-B1 is the primary receptor for high-density lipoproteins, while CD36 is the receptor responsible for the internalization of oxidized low-density lipoproteins. Despite their importance, class B scavenger receptor structure has only been studied by functional domain or peptide fragments—there are currently no reports of utilizing purified full-length protein. Here we report the successful expression and purification of full-length human SR-B1 and CD36 using an *Spodoptera frugiperda* insect cell system. We demonstrate that both SR-B1 and CD36 retained their normal functions in *Spodoptera frugiperda* cells, including lipoprotein binding, lipid transport, and the formation of higher order oligomers in the plasma membrane. Purification schemes for both scavenger receptors were optimized and their purity was confirmed by SDS-PAGE. Both purified scavenger receptors were assessed for stability by thermal shift assay and shown to maintain stable melting temperatures up to 6 weeks post-purification. Microscale thermophoresis was used to demonstrate that purified SR-B1 and CD36 were able to bind their native lipoprotein ligands. Further, there was no difference in affinity of SR-B1 for high-density lipoprotein or CD36 for oxidized low-density lipoprotein, when comparing glycosylated and deglycosylated receptors. These studies mark a significant step forward in creating physiologically relevant tools to study scavenger receptor function and lay the groundwork for future functional studies and determination of receptor structure.

Atherosclerosis is the major cause of cardiovascular disease, the leading cause of death globally since the early 1900s (1). Atherosclerotic plaques consisting of fat-accumulating macrophages, cellular debris, cholesterol, lipids, and lipoproteins build up in arteries and can lead to acute medical events like myocardial infarction and stroke. While many factors contribute to atherosclerotic plaque formation and disease, the balance between lipoprotein-cholesterol deposition and clearance is an important factor that impacts plasma lipid control and disease risk (2). Two members of the class B scavenger receptor family, cluster of differentiation 36 (CD36) and scavenger receptor class B type 1 (SR-B1), play central roles in lipoprotein metabolism and have been implicated in atherosclerosis. CD36 is the primary receptor for oxidized low-density lipoproteins (oxLDLs) and facilitates the unregulated deposition of cholesterol into macrophages to initiate foam cell formation and promote atherosclerotic plaque development (3). On the other hand, SR-B1 plays a protective role against atherosclerosis, as it serves as the primary receptor for high-density lipoproteins (HDLs) (4) and facilitates bidirectional cholesterol transport. SR-B1 mediates the efflux of free cholesterol from peripheral cells into HDL (5), as well as the selective uptake of cholesteryl esters within HDL into cells (6, 7). The interaction of SR-B1 and HDL on the surface of the liver is crucial in the final steps of the reverse cholesterol transport pathway, which is the body’s primary mechanism to recycle or excrete plasma cholesterol. The importance of the lipid-modulating effects of class B scavenger receptors is well demonstrated in humans, as patients with variations in the genes encoding SR-B1 or CD36 have dyslipidemia and increased cardiovascular disease risk (reviewed in (8, 9)).

While CD36 and SR-B1 play opposing roles in atherosclerotic disease progression, they share a common structural topology consisting of cytoplasmic N- and C-terminal tails, two anchoring transmembrane domains, and a large extracellular domain. The extracellular domains are responsible for binding ligands and are the most studied region of the class B scavenger receptors. The structures of the extracellular regions of human CD36 and another class B scavenger receptor, lysosomal integral membrane protein 2, were resolved by X-ray crystallography (10, 11, 12), representing a major step forward in our understanding of scavenger receptor structure. Despite this emphasis on the extracellular regions, critical functions of SR-B1 and CD36 are also mediated by the transmembrane domains and intracellular tails. In addition to the importance of transmembrane domains in facilitating lipid transport (13, 14, 15), both CD36 and SR-B1 have been shown to homo-oligomerize through interactions of their transmembrane domains (16, 17, 18, 19, 20). Currently, the only existing structural information about class B scavenger receptor transmembrane regions is the NMR structure of an SR-B1 peptide spanning the C-terminal transmembrane domain and a flanking extracellular region (20). This C-terminal transmembrane domain has also been shown to mediate SR-B1’s membrane cholesterol-sensing function (21). The C-terminal cytoplasmic domains of CD36 and SR-B1 are also important as they mediate downstream signaling cascades that help regulate cellular lipid metabolism (22, 23). Previous work has relied on mutagenesis studies to identify these critical regions (reviewed in (8, 24)), as a full-length structure of any class B scavenger receptor has not yet been resolved.

Here, we present data illustrating our ability to express and purify functional full-length human CD36 and SR-B1. Our studies represent a shift from the simplicity of previous peptide studies toward analyzing full-length functional membrane proteins in lipid reconstitution systems. Full-length protein is a necessary tool that allows us to develop structurally guided hypotheses to accurately study how these receptors function in the human body.

## Results

### SR-B1 and CD36 express at the surface of Sf9 cells

**Figure 1:**
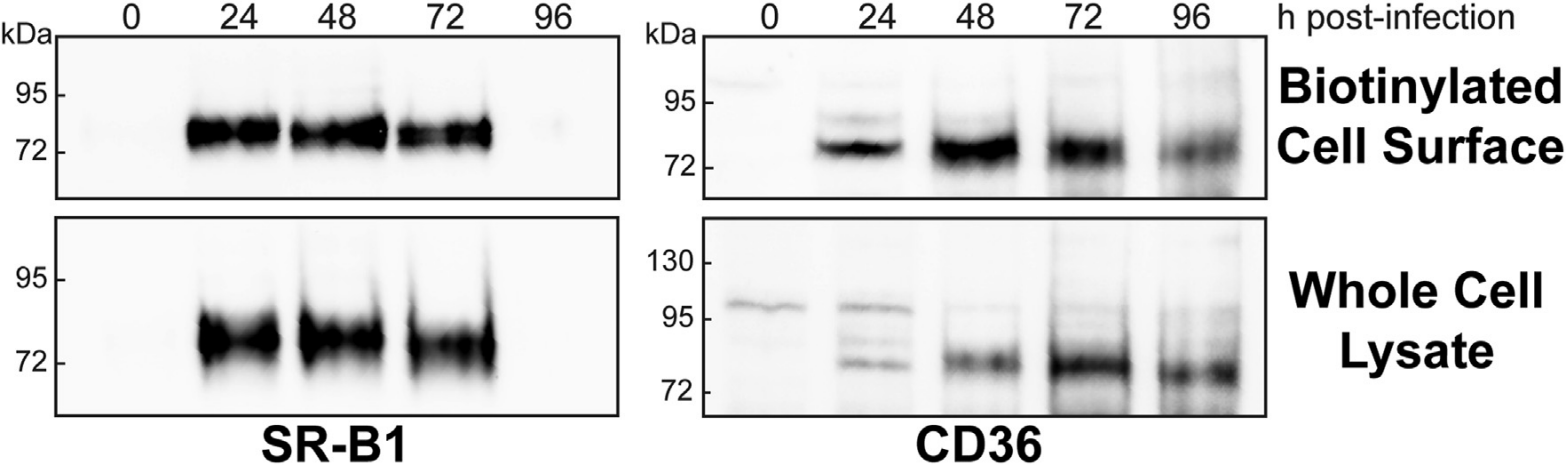
**SR-B1 and CD36 express at the surface of Sf9 cells.** Lysates from Sf9 cells infected with SR-B1– or CD36-expressing baculovirus at an MOI of 5 were collected every 24 h post-infection, following incubation with NHS-biotin as described in Experimental procedures. Immunoblot analysis of 40 μl of biotinylated cell surface proteins (*top panels*) or 15 μg of whole-cell lysates (*bottom panels*) was performed and receptors were detected using an antibody directed against SR-B1 (approximately 82 kDa) or CD36 (approximately 88 kDa). Immunoblots are representative of four independent infections. SR-B1, scavenger receptor class B type 1; CD36, cluster of differentiation 36; MOI, multiplicity of infection; Sf9, *Spodoptera frugiperda*.

Purified human full-length proteins are essential tools to better understand the functions of class B scavenger receptors and their roles in health and disease. We selected a *Spodoptera frugiperda* (Sf9) insect cell/baculoviral system to express our proteins of interest as they possess the post-translational modification machinery often required for protein expression and function. Sf9 cells were infected with recombinant baculovirus encoding human full-length SR-B1 or human full-length CD36 at a multiplicity of infection (MOI) of 5 to induce robust protein expression. Lysates were collected in 24 h increments post-infection. To verify expression, immunoblots were performed to detect human SR-B1 or human CD36. At 0 h, the lack of bands suggest that Sf9 cells do not endogenously express detectable levels of SR-B1 or CD36. Expression of both SR-B1 and CD36 increased up to 72 h postinfection in whole-cell lysates (Fig. 1, bottom panels). Cell surface expression of CD36 and SR-B1 was assayed by lysine-linked, membrane-impermeable biotinylation and subsequent streptavidin immunoprecipitation. Both CD36 and SR-B1 express at the cell surface, with expression peaking at 48 h postinfection (Fig. 1, top panels). Lower bands in CD36 immunoblots may be due to nonspecific interaction with insect cell proteins. After 96 h, baculoviral infection is lethal to cells (25) and may account for the changes in whole cell and cell surface expression at later time points. Due to these patterns in expression, all functional assays performed in plated cells were performed no later than 72 h post-infection.

### SR-B1 and CD36 expressed in Sf9 cells bind lipoproteins and transport lipid

**Figure 2:**
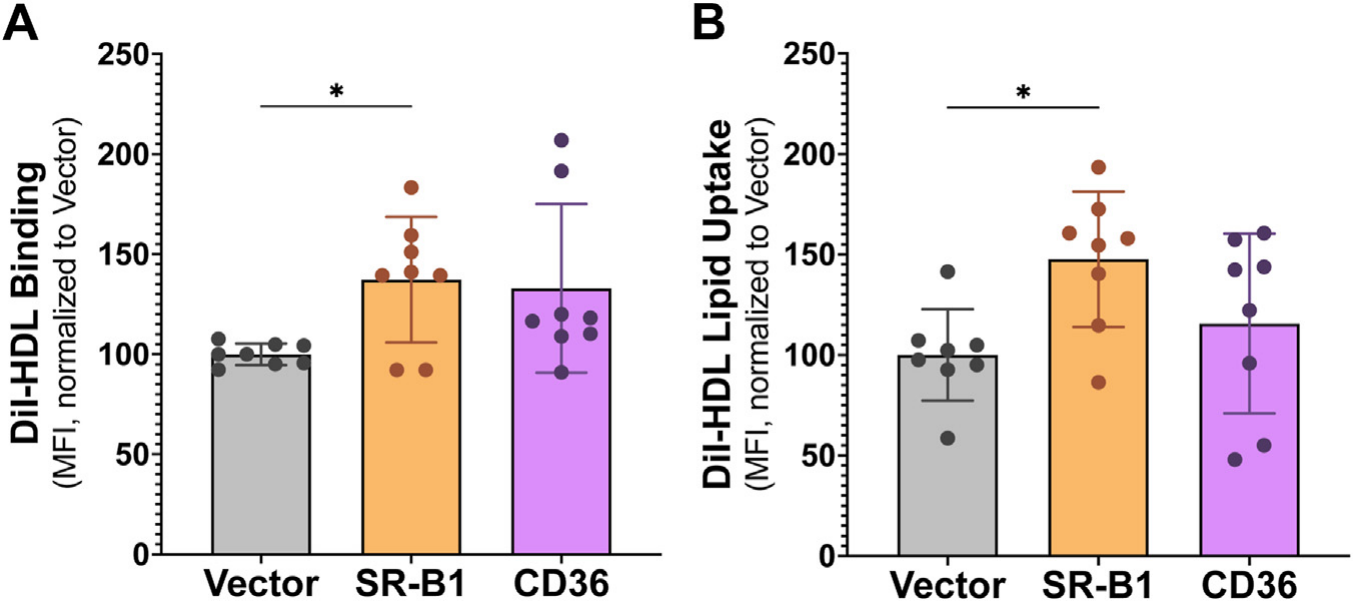
**SR-B1–expressing Sf9 cells mediate DiI-HDL binding and DiI-lipid uptake from HDL.** Sf9 cells were infected with high-titer baculovirus encoding SR-B1, CD36, or empty vector at an MOI of 5 and immediately plated in 12-well plates. At 72 h post-infection, cells were washed and incubated with 10 μg/ml DiI-HDL in media + 0.5% BSA for 90 min at 4 °C to assess binding (*A*) or at 27 °C to assess combined binding and uptake. After 90 min, cells were harvested in PBS/0.5% BSA. MFI was recorded by flow cytometry and DiI uptake (*B*) was calculated by subtracting the MFI at 4 °C from the MFI at 27 °C. Data were analyzed by one-way ANOVA and Dunnett’s multiple comparisons, presented as mean ± SD, n = 4 independent infections, with technical replicates performed in duplicate. ∗*p* ≤ 0.05. BSA, bovine serum albumin; CD36, cluster of differentiation 36; DiI, 1,1′-dioctadecyl-3,3,3′,3′-tetramethylindocarbocyanine; HDL, high-density lipoprotein; MOI, multiplicity of infection; MST, microscale thermophoresis; Sf9, *Spodoptera frugiperda*; SR-B1, scavenger receptor class B type 1.

**Figure 3:**
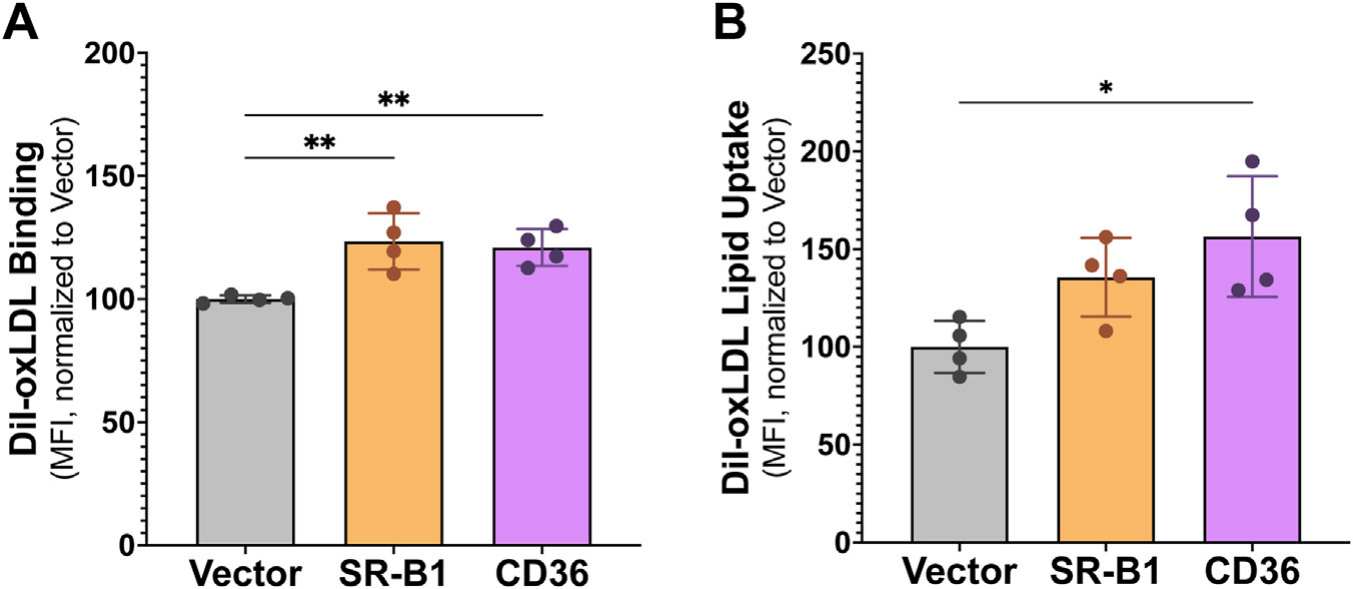
**Sf9 cells expressing CD36 increase DiI-oxLDL binding and uptake.** Sf9 cells were infected with high-titer baculovirus encoding SR-B1, CD36, or empty vector at an MOI of 5 and immediately plated in 12-well plates. At 72 h post-infection, cells were washed and incubated with 10 μg/ml DiI-oxLDL in media + 0.5% BSA for 90 min at 4 °C to assess binding (*A*) or at 27 °C to assess combined binding and uptake. After 90 min, cells were harvested in PBS/0.5% BSA. MFI was recorded by flow cytometry and DiI uptake (*B*) was calculated by subtracting the MFI at 4 °C from the MFI at 27 °C. Data were analyzed by one-way ANOVA and Dunnett’s multiple comparisons, presented as mean ± SD, n = 2 independent infections, with technical replicates performed in duplicate. ∗*p* ≤ 0.05 and ∗∗*p* ≤ 0.01. BSA, bovine serum albumin; CD36, cluster of differentiation 36; DiI, 1,1′-dioctadecyl-3,3,3′,3′-tetramethylindocarbocyanine; MFI, mean fluorescence intensity; MOI, multiplicity of infection; oxLDL, oxidized low-density lipoprotein; Sf9, *Spodoptera frugiperda*; SR-B1, scavenger receptor class B type 1.

With robust expression confirmed, our next step was to verify that the two class B scavenger receptors of interest maintained their expected functions when expressed in Sf9 cells. The incorporation of the fluorescent lipid, 1,1′-dioctadecyl-3,3,3′,3′-tetramethylindocarbocyanine perchlorate (DiI), into lipoprotein particles allows us to measure lipoprotein binding and lipid uptake by flow cytometry. At 72 h post-infection, Sf9 cells were treated with 10 μg/ml DiI-HDL or DiI-oxLDL for 90 min at either 4 °C or 27 °C and the mean fluorescence intensity (MFI) of DiI-lipoprotein binding alone (4 °C) or combined binding and DiI-lipid uptake (27 °C) was measured by flow cytometry. We show that expression of SR-B1 in Sf9 cells significantly increased DiI-HDL binding (Fig. 2*A*) and DiI-lipid uptake (Fig. 2*B*) by 37% and 48%, respectively, compared to cells infected with empty vector. CD36 was also able to bind DiI-HDL and mediate DiI-HDL-lipid uptake, however, the differences compared to empty vector were not statistically significant. When DiI-oxLDL binding and uptake were assessed, CD36 facilitated a 21% increase in oxLDL binding (Fig. 3*A*) and an 56% increase in uptake (Fig. 3*B*) compared to empty vector–infected cells. Interestingly, despite a statistically significant increase in the ability of SR-B1 to bind DiI-oxLDL (Fig. 3*A*), this receptor was unable to mediate delivery of oxLDL DiI-lipid into Sf9 cells (Fig. 3*B*).

### SR-B1 and CD36 form dimers and higher order oligomers in Sf9 cell membranes

**Figure 4:**
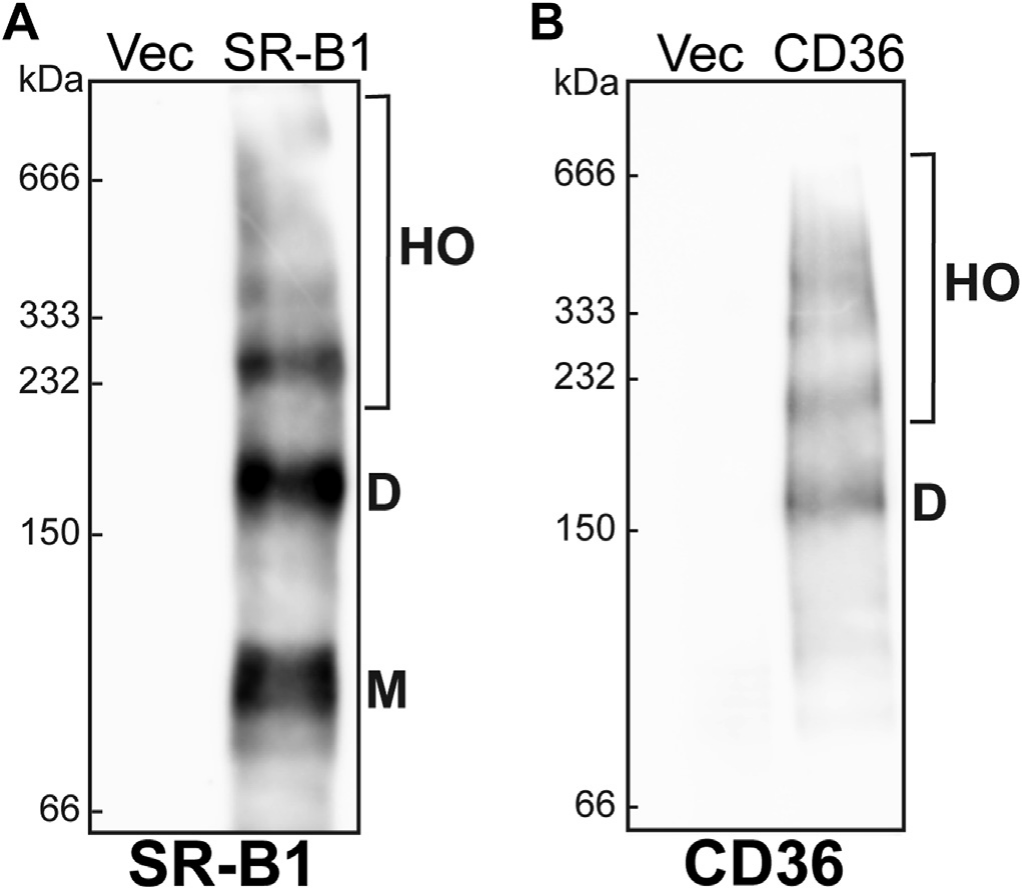
**SR-B1 and CD36 form higher order oligomers in Sf9 Cells.** Cell lysates from empty vector (Vec), SR-B1-, or CD36-baculovirus-infected Sf9 cells were collected at 72 h post-infection. Lysates were sonicated and separated by nondenaturing perfluorooctanoic acid (PFO)-PAGE on 8% polyacrylamide gels. Immunoblot analysis utilized an antibody against SR-B1 (*A*) or CD36 (*B*) was used to detect the formation of monomers (M), dimers (D), and higher order oligomers (HO). Data are representative of immunoblots of lysates from three independent infections. CD36, cluster of differentiation 36; Sf9, *Spodoptera frugiperda*; SR-B1, scavenger receptor class B type 1.

SR-B1 has been shown to form dimers and higher order oligomers in diverse cell and tissue types (16, 18). When a leucine zipper dimerization motif is mutated in the C-terminal region of SR-B1, higher order homo-oligomers do not form and SR-B1 loses its ability to bidirectionally transport cholesterol (5, 6, 7). CD36 has also been shown to oligomerize (26), but the role of oligomerization in function remains poorly understood. Our data indicate that both SR-B1 (Fig. 4*A*) and CD36 (Fig. 4*B*) maintain the ability to form oligomers in Sf9 cells as detected by perfluorooctanoic acid (PFO)-PAGE.

### Full-length SR-B1 and CD36 can be purified from Sf9 cells

**Figure 5:**
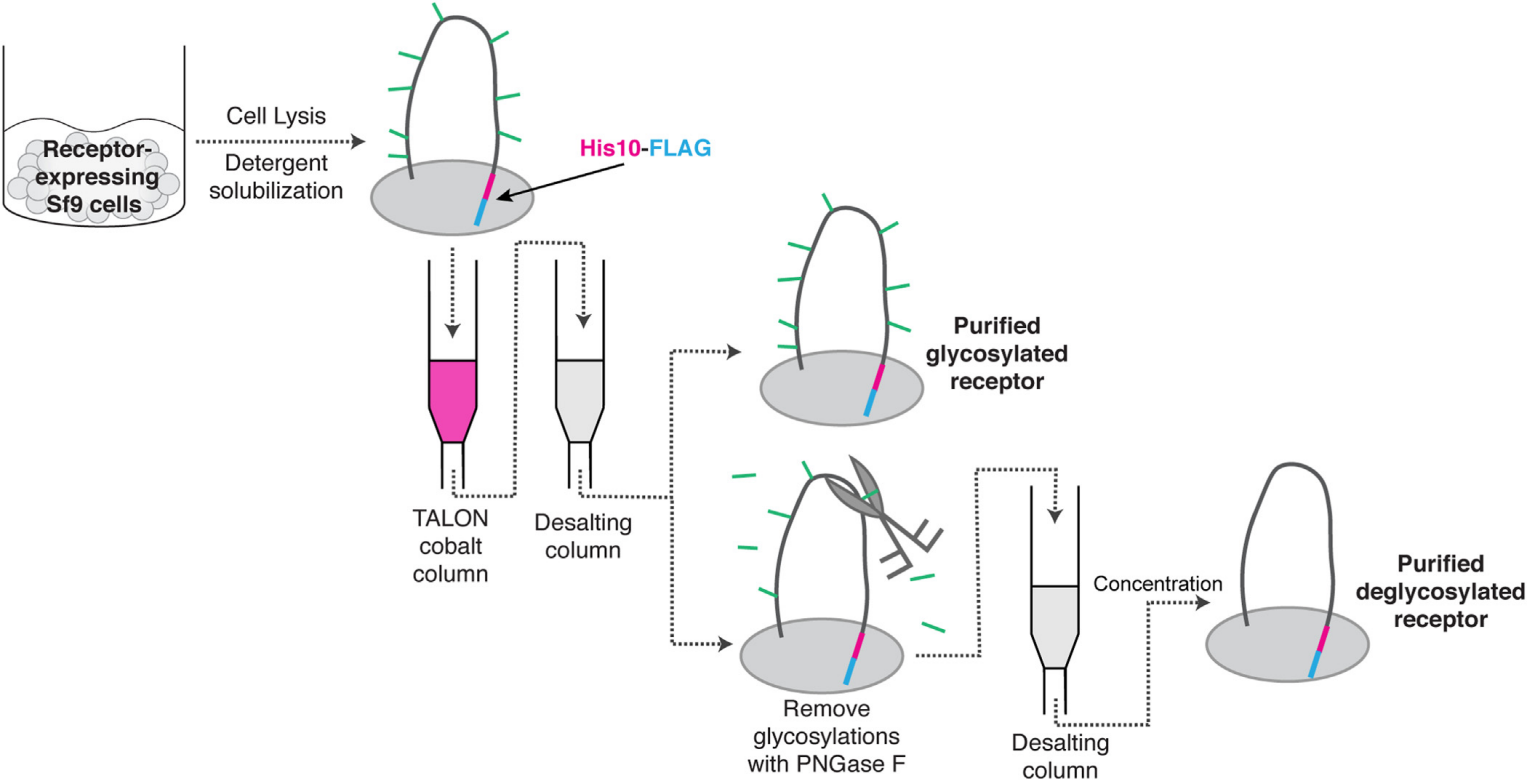
**Schematic representation of scavenger receptor purification protocol.** Sf9 cells were infected with baculovirus encoding SR-B1 or CD36, each flanked on the C terminus by a H10 histidine and FLAG tag. Lysates were solubilized with LMNG/CHS detergent (final concentration: 0.025% LMNG, 0.005% CHS) and tagged proteins were enriched by flow through a TALON cobalt affinity column. Tags and glycosylations can then be enzymatically removed. Final contaminants were removed by an additional flow through an affinity and desalting column. CD36, cluster of differentiation 36; CHS, cholesteryl hemisuccinate; LMNG, lauryl maltose neopentyl glycol; Sf9, *Spodoptera frugiperda*; SR-B1, scavenger receptor class B type 1.

**Figure 6:**
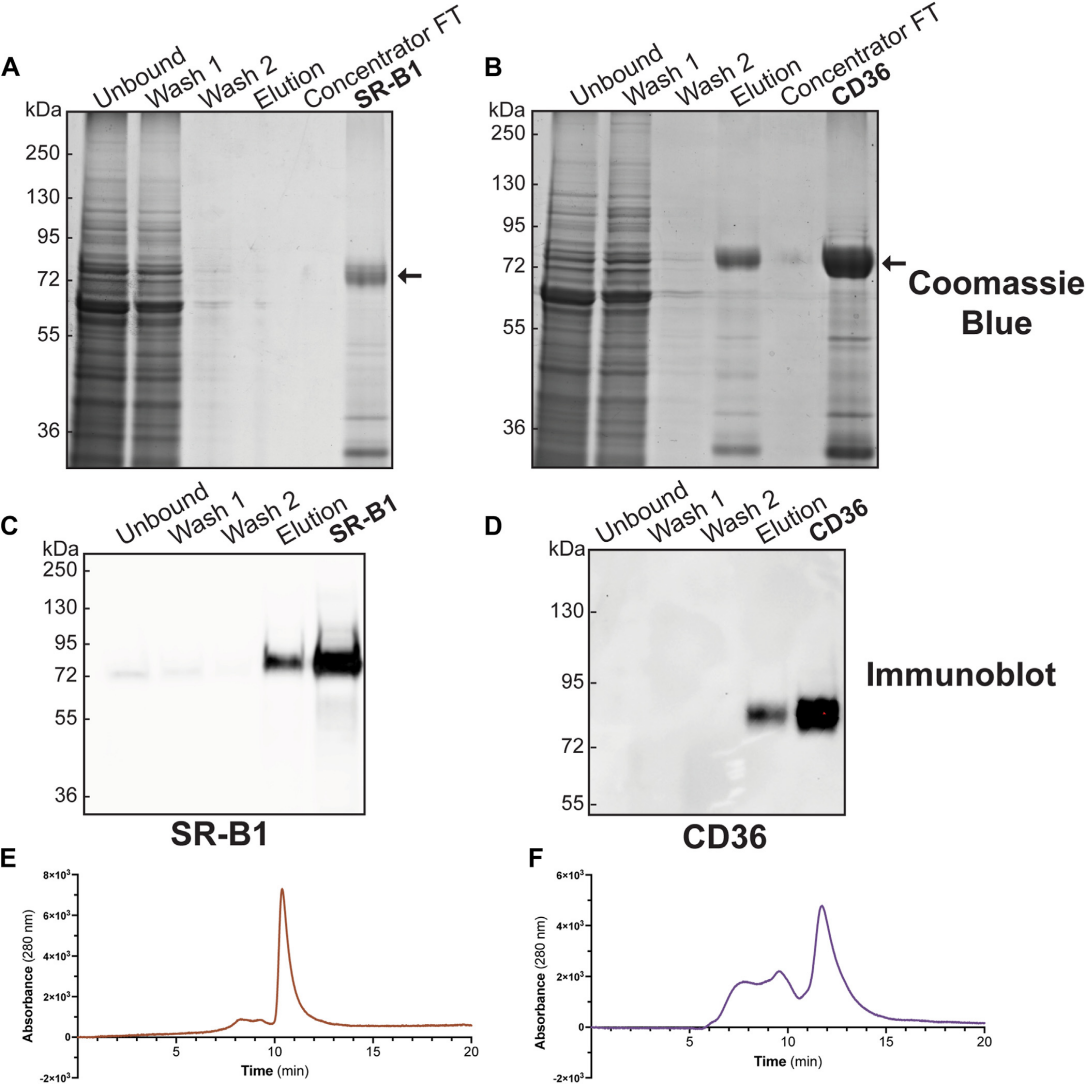
**Purification protocol yields pure SR-B1 and CD36.** Samples (∼15 μl) were collected at various points in the purification protocol and were separated by SDS-PAGE. Sample quality was verified by *Coomassie blue* (*A* and *B*) and immunoblot analysis (*C* and *D*). Size-exclusion chromatography for SR-B1 (*E*) or CD36 (*F*) was also performed. Blots are representative of four independent purifications. CD36, cluster of differentiation 36; FT, flow-through; SR-B1, scavenger receptor class B type 1.

Having verified that both SR-B1 and CD36 maintain expected lipid transport functions and oligomeric properties when expressed in Sf9 cells, we wanted to leverage this system to express and purify full-length receptors. To do so, Sf9 cells in their logarithmic growth phase were infected with baculovirus encoding human CD36 or human SR-B1 at an MOI of 5. Cells were grown for 72 h and an extensive purification protocol was employed. As shown in Figure 5, lysates were processed with a modified membrane preparation in which a lauryl maltose neopentyl glycol (LMNG)/cholesteryl hemisuccinate (CHS) detergent mixture was used to solubilize membranes and cells were subsequently syringe-lysed and centrifuged at 50,000*g* for 30 min at 4 °C. Resultant clarified solubilized membranes were incubated with TALON cobalt affinity resin, washed, and eluted with buffer containing imidazole. Samples were desalted to remove imidazole and concentrated. This purification scheme resulted in pure protein solubilized in detergent micelles (final concentrations of 0.025% LMNG, 0.005% CHS), shown by sampling through steps of our purification process and separating samples by SDS-PAGE and staining with Coomassie blue (Fig. 6, *A* and *B*). Pure protein yields per 1 liter of cell culture are approximately 0.5 mg for SR-B1 and 1 mg for CD36. Sample purity was further verified *via* immunoblot analysis using antibodies directed against either human CD36 or SR-B1 (Fig. 6, *C* and *D*). As part of the purification process, size-exclusion chromatography (SEC) was performed to assess the size and homogeneity of SR-B1 and CD36 (Fig. 6, *E* and *F*). SR-B1 elutes primarily as a large peak around 10.5 min, while CD36’s largest peak elutes around 12 min. Both proteins, but more so CD36, have a double-humped peak that elutes prior to the most intense peak.

### Purified SR-B1 and CD36 retain stable melting temperatures after purification

**Figure 7:**
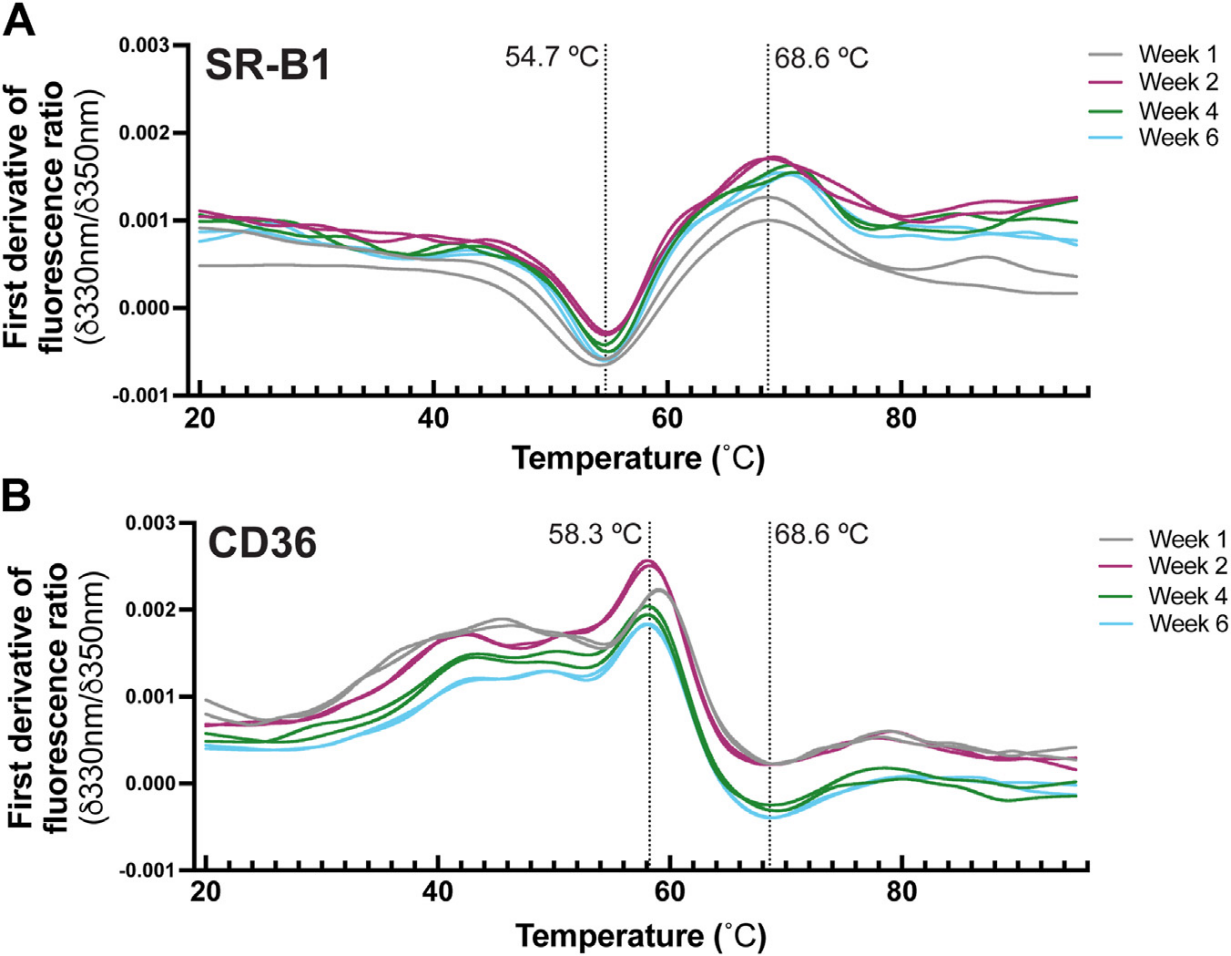
**SR-B1 and CD36 remain stable up to 6 weeks post-purification.** Purified SR-B1 and CD36 stability was measured up to 6 weeks post-purification by Prometheus NT.48. Protein unfolding as a function of increasing temperature was monitored by assessing changes in intrinsic tryptophan fluorescence at 330 and 350 nm at 20% power over a temperature gradient from 20 ˚C to 90 ˚C. Destabilization events for SR-B1 in detergent micelles occurred at 54.7 ˚C and 68.6 ˚C (*A*) and at 58.3 ˚C and 68.6 ˚C for CD36 (*B*). The destabilization temperatures were calculated by taking the ratio of the first derivative values of the sample fluorescence at 350 and 330 nm. Data represent two readings per construct per time point. Details of the thermal shift assay can be found in Experimental procedures. CD36, cluster of differentiation 36; SR-B1, scavenger receptor class B type 1.

Our next goal was to verify that purified SR-B1 and CD36 were stable in detergent micelles over time, when stored at 4 °C. To do this, we utilized a thermal shift assay to monitor sample destabilization or unfolding over an increasing temperature gradient by measuring the intrinsic tryptophan fluorescence of proteins as described in Experimental procedures. Figure 7 demonstrates that both SR-B1 and CD36 maintained stable melting temperatures of 54.7 °C and 58.3 °C, respectively, illustrating that purified SR-B1 and CD36 can be reliably used for experiments for up to 6 weeks postpurification.

### Purified SR-B1 and CD36 bind their native ligands

Microscale thermophoresis (MST) allows us to assess binding affinities of labeled receptors to various ligands (27) and is described in detail in Experimental procedures. Both CD36 and SR-B1 were labeled with Cy5 (*via* attachment to lysine residues) and incubated with a variety of ligands known to bind these receptors. Of note, resultant K_d_ values from MST experiments are most accurately reported in μg/ml concentrations rather than molarity. Lipoprotein species are generally difficult to characterize by molecular weight due to their heterogenous compositions and are instead typically characterized by their relative density. However, using average molecular weights of 267.5 kDa for HDL and 3500 kDa for oxLDL, we can report an apparent K_d_ in molarity, but the caveats must be considered.

**Figure 8:**
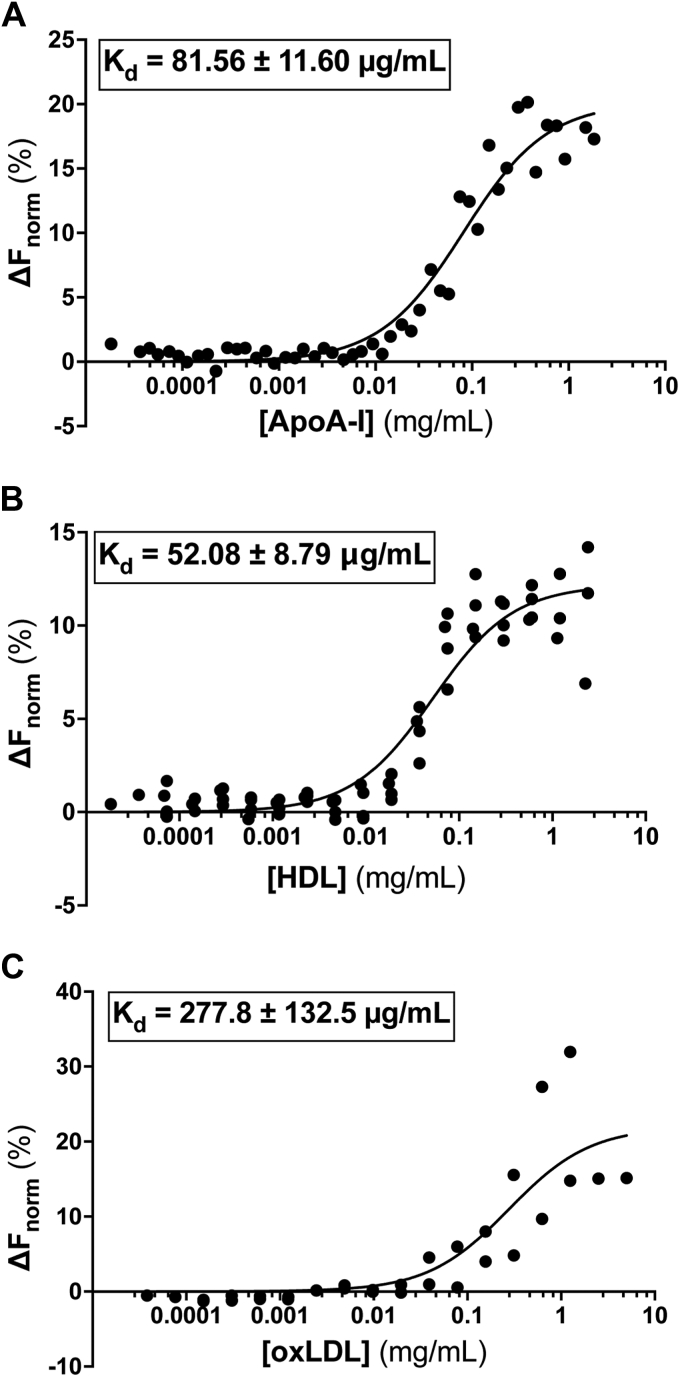
**Purified SR-B1 binds to native ligands.** MST was used to assess binding of Cy5-labeled SR-B1 to various ligands. Measurements were performed on the Monolith NT.115 BLUE/RED at 25 °C using 40% MST power and laser on/off times of 0 and 21 s. Each point is representative of a single point in a 16-point titration. Apparent K_d_ values for glycosylated SR-B1 binding to apoA-I (*A*, R^2^ = 0.951), HDL (*B*, R^2^ = 0.908), and oxLDL (*C*, R^2^ = 0.749) were calculated by nonlinear regression analysis, assuming one-site specific binding, of the normalized thermophoresis at increasing ligand concentrations, using GraphPad Prism. Curves are representative of at least two independent purifications, ligand preparations, and labelings. Further information about MST methods can be found in Experimental procedures. HDL, high-density lipoprotein; MST, microscale thermophoresis; oxLDL, oxidized low-density lipoprotein; SR-B1, scavenger receptor class B type 1.

**Figure 9:**
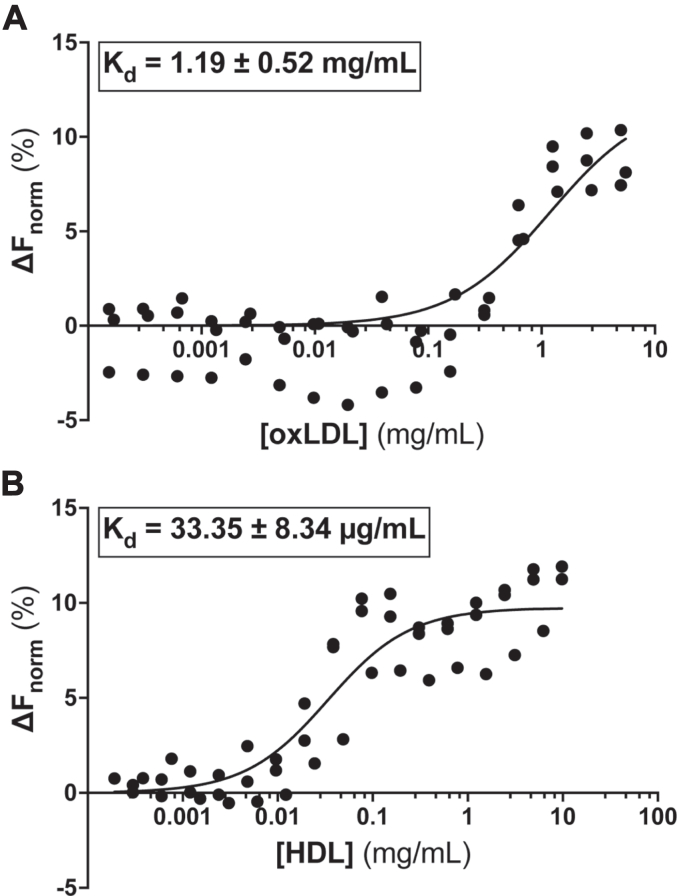
**Purified CD36 binds to oxLDL and HDL.** MST was used to assess binding of Cy5-labeled SR-B1 to various ligands. Measurements were performed on the Monolith NT.115 BLUE/RED at 25 °C using 40% MST power and laser on/off times of 0 and 21 s. Each point is representative of a single point in a 16-point titration. Apparent K_d_ values for glycosylated CD36 binding to oxLDL (*A*, R^2^ = 0.765) and HDL (*B*, R^2^ = 0.845) were calculated by nonlinear regression analysis, assuming one-site specific binding, of the normalized thermophoresis at increasing ligand concentrations, using GraphPad Prism. Curves are representative of at least two independent purifications, ligand preparations, and labelings. Further information about MST methods can be found in Experimental procedures. CD36, cluster of differentiation 36; HDL, high-density lipoprotein; MST, microscale thermophoresis; oxLDL, oxidized low-density lipoprotein; SR-B1, scavenger receptor class B type 1.

Using MST, we demonstrate that SR-B1 binds to lipid-free apolipoprotein A-I (apoA-I), the main protein component of HDL, with a calculated K_d_ of 82 ± 12 μg/ml or 2.73 ± 0.40 μM (Fig. 8*A*). As expected, SR-B1 binds to holoparticle HDL, its preferred ligand, with a lower K_d_ of 52 ± 9 μg/ml or 194 ± 34 nM (Fig. 8*B*). The increase in affinity when binding HDL compared to apoA-I is consistent with previously published literature (28). SR-B1 has also been implicated in binding other lipoproteins with varying affinities. This cross-reactivity is demonstrated in our cellular assays in Figures 2 and 3 and is now supported by MST. Figure 8*C* illustrates that purified human SR-B1 also binds oxLDL, albeit with much lower affinity than to HDL. We used the same technique to assess CD36’s ligand-binding affinities. CD36 is a known receptor for oxLDL and MST assays demonstrate that purified CD36 binds oxLDL with an affinity of 1.2 ± 0.5 mg/ml or 342.86 ± 285.71 nM (Fig. 9*A*). CD36 also binds human HDL, but with a higher affinity (K_d_ of 33 ± 8 μg/ml or 123.36 ± 29.9 nM) than for oxLDL (Fig. 9*B*).

### Glycosylation state of SR-B1 and CD36 do not impact ligand binding affinity

**Figure 10:**
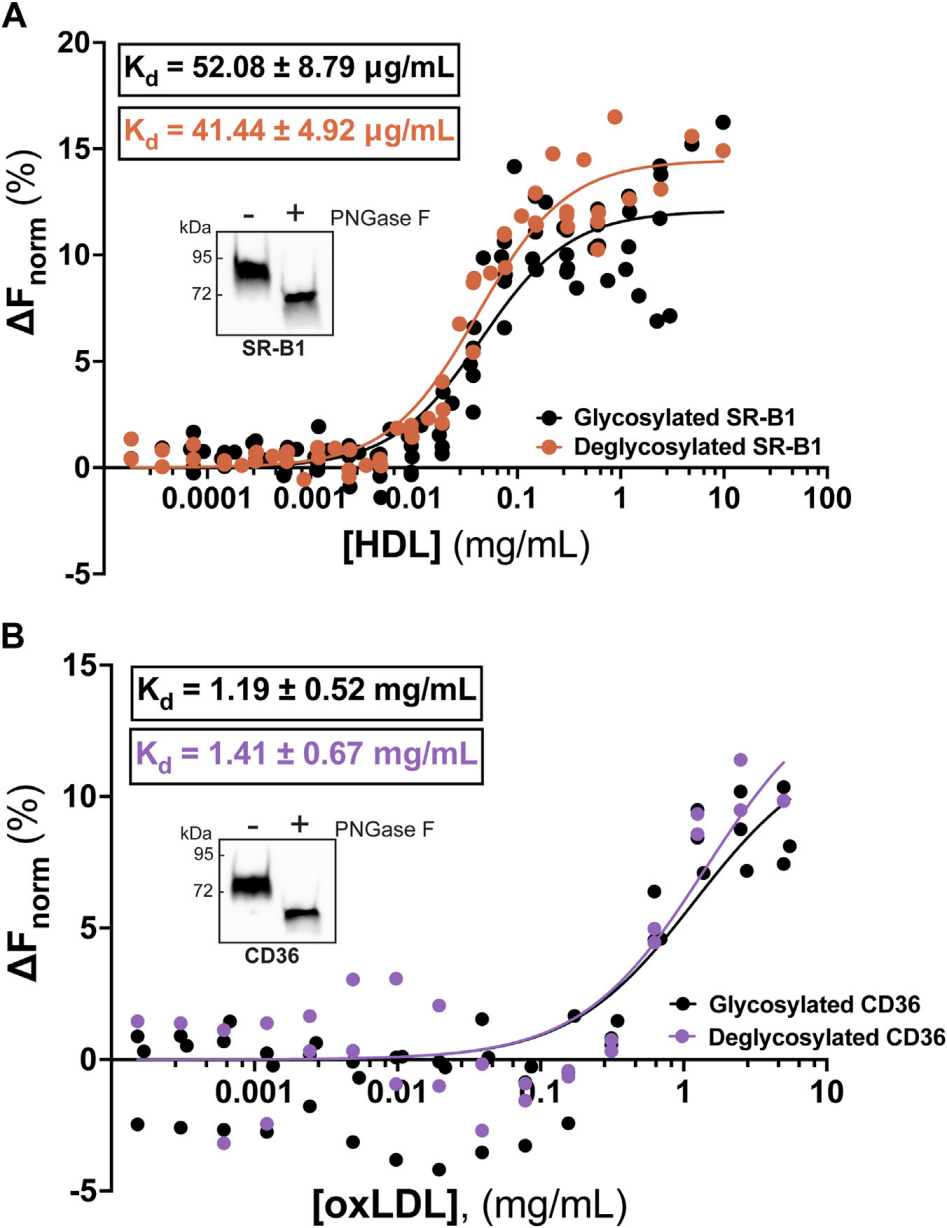
**Glycosylation status of SR-B1 and CD36 does not impact ligand binding.** Human full-length CD36 or SR-B1 were deglycosylated by PNGase F, as demonstrated by differences in migration by SDS-PAGE (insets). Proteins were subsequently labeled with Cy5 for MST experiments and performed as described in Figures 7 and 8. Apparent K_d_ values for deglycosylated SR-B1 binding to HDL (*A*, R^2^ = 0.952) and deglycosylated CD36 binding to oxLDL (*B*, R^2^ = 0.799) were calculated by nonlinear regression analysis, assuming one-site specific binding, of the normalized thermophoresis at increasing ligand concentrations, using GraphPad Prism. MST curves of glycosylated proteins were calculated by nonlinear regression analysis, assuming one-site specific binding, of the normalized thermophoresis at increasing ligand concentrations, using GraphPad Prism. Curves are representative of at least two independent purifications, ligand preparations, and labelings. Further information about MST methods can be found in Experimental procedures. CD36, cluster of differentiation 36; HDL, high-density lipoprotein; MST, microscale thermophoresis; oxLDL, oxidized low-density lipoprotein; SR-B1, scavenger receptor class B type 1.

It has been well established that glycosylation is required for SR-B1 and CD36 to express at the cell surface (29, 30). An advantage of our purification system is that it allows for the study of purified protein in both glycosylated and deglycosylated states, as shown in Figure 5. To deglycosylate proteins, samples were incubated with PNGase F overnight and concentrated with a 50 kDa molecular weight cut-off (MWCO) spin concentrator to remove the enzyme. When comparing the MST profiles of our glycosylated and deglycosylated SR-B1 preparations, no significant differences were observed in K_d_ values for HDL (Fig. 10*A*). The same trend is seen with CD36, where glycosylation status does not impact the oxLDL-binding affinity (Fig. 10*B*).

## Discussion

All currently existing structural information about class B scavenger receptors has been obtained using mutagenesis strategies, extracellular domain-only constructs, and short peptides. While these are helpful tools in beginning to understand structure-function relationships, a full-length structure of SR-B1 or CD36 would greatly enhance our understanding of how these receptors function in both healthy and disease states. The current report addresses this gap and sets the stage for high-resolution structural determination using novel tools.

Our studies demonstrate that SR-B1 and CD36 can be expressed in Sf9 cells using a recombinant baculovirus infection system to induce scavenger receptor expression. Both receptors were shown to express at the cell surface and displayed their expected functions in the Sf9 plasma membrane—namely lipoprotein binding and lipid uptake. There are several reasons why the fold changes observed in lipoprotein binding and lipid uptake are not as dramatic in the insect cell system compared to previously reported values obtained from mammalian cell transient transfection models. First, decreased activity of membrane proteins expressed within Sf9 cells is not uncommon (31) and may be explained by the unique lipid composition of Sf9 cell membranes (32, 33). Specifically, Sf9 cells have a lower cholesterol:phospholipid ratio and no ability to esterify cholesterol (33). As both SR-B1 and CD36 are extensively involved in lipid homeostasis and transport, it is unsurprising that these variations in membrane and cellular lipid makeup would impact their functionality. The binding and uptake assays are well validated and have been performed in many other mammalian systems (3, 34, 35, 36, 37, 38, 39) and suggest that effects seen are insect cell–specific. Additionally, many have postulated that lipid transport by class B scavenger receptors requires protein partners (23, 40, 41). In the absence of other mammalian proteins in insect cell membranes, scavenger receptor function could be reduced. Moreover, Sf9 cells, while capable of N-linked glycosylation, attach carbohydrate modifications of lower complexity than the mammalian glycosylation machinery (42). The role of glycosylation, especially complex glycosylation, in SR-B1 and CD36 function remains understudied but may contribute to discrepancies between Sf9 and mammalian cell data. Lastly, Sf9 cells are cultured in ambient CO_2_ at 27 °C, while mammalian cells are usually maintained at 5% CO_2_ and 37 °C. As such, uptake assays, performed at 37 °C in mammalian systems, must be carried out at 27 °C to keep the Sf9 cells alive. This difference in temperature likely impacts the efficiency of lipid transport and may account for lower fold changes seen in lipid uptake upon scavenger receptor expression in Sf9 cells. The goal of these experiments was to verify that these proteins retained function in an exogenous system, which is clearly demonstrated by an increase in HDL and oxLDL binding and lipid uptake when comparing SR-B1– and CD36-expressing cells to empty vector–infected cells, respectively.

Both CD36 and SR-B1 have been shown to homo-oligomerize in various cell and tissue types (18, 19, 26, 43). We were able to visualize oligomer formation of both CD36 and SR-B1 in Sf9 cell membranes using PFO-PAGE, a modified electrophoresis technique which uses PFO in place of SDS to preserve native membrane interactions. We also utilized SEC to characterize additional biophysical properties of our protein products. Both SR-B1 and CD36 first elute as a double-humped peak, followed by the most intense peak. It is possible that these peaks represent oligomeric forms of each protein, but future studies are required to delineate the precise composition and mass of these peaks. While exact oligomeric states and the role of oligomerization in receptor function remain understudied, these results demonstrate that receptor homo-oligomerization is independent of cell system or interacting proteins (44). Oligomerization motifs have been identified within the transmembrane domains of both SR-B1 and CD36 (19, 20, 26, 45), but little is known about the role of these motifs in oligomeric complex formation, providing an avenue for continued exploration. Future studies of oligomeric properties of purified proteins can be performed with electron microscopy, dynamic light scattering, crosslinking agents, or native PAGE to gain valuable insight into the mechanisms of oligomerization of class B scavenger receptors and the impact of oligomer formation on lipid transport functions.

After verifying scavenger receptor function in plated cells, we were successful in purifying full-length human SR-B1 and CD36 from Sf9 cells. Coomassie blue–stained SDS-PAGE gels and immunoblots showed that our protocol yields a pure and stable protein product suitable for downstream functional and structural studies. This is the first published report of successful purification of full-length human class B scavenger receptors. Others have solved partial structures of scavenger receptors, including the extracellular domain of human CD36 (10), the C-terminal transmembrane domain and flanking extracellular region of mouse SR-B1 (20), and the extracellular region of lysosomal integral membrane protein 2 (11, 12). We have previously combined this structural data with computational prediction to generate homology models of SR-B1 and CD36 (8, 24).

To validate that purified receptors maintained their biological functions, we assayed native ligand binding capabilities of purified proteins by MST. When we calculated K_d_ values using pure protein and ligands, the trends in K_d_ values aligned closely to what has been previously shown experimentally. Specifically, SR-B1 binds with tighter affinity to holoparticle HDL than delipidated apoA-I. We also observed that CD36 binds HDL with higher affinity than SR-B1, and SR-B1 binds oxLDL with higher affinity than CD36. These trends in receptor-ligand affinity have been previously reported using other binding assay techniques (3, 46) and we now verify the same patterns by MST analysis. Interestingly, for the SR-B1/HDL interaction, we calculated a K_d_ value of 52 μg/ml, while Rodrigueza *et al*. reported a K_d_ of 8.7 μg/ml (47). Similarly, CD36 and oxLDL have been reported to bind with a K_d_ ranging from 1.50 to 10.44 μg/ml, while we calculated a K_d_ of 1.19 mg/ml (3, 46). There are several possible reasons for the discrepancies in reported values. Previous studies utilized murine receptor constructs, lipoproteins isolated from mice, radiolabeled lipoproteins, or even recombinant lipoprotein particles, while our studies use human scavenger receptor constructs, as well as human lipoprotein ligands (4, 44, 46, 47, 48, 49). Further, we are the first to publish lipoprotein binding data using the novel MST technique and there may be additional intrinsic constraints introduced by this platform (reviewed in (27)). Our MST sample preparation requires labeling SR-B1 and CD36 proteins with Cy5 *via* lysine residues, which may obstruct binding interfaces and affect affinity. The large size and diverse composition of lipoproteins likely influence the K_d_ measurements obtained using MST. For example, the calculated affinity of oxLDL for CD36 was surprisingly low and could be the result of the extremely large size of oxLDL, with average diameters of 150 nm compared to 10 nm for HDL. Most importantly, for the purpose of this study, our purified proteins still demonstrate robust and reproducible ligand binding capabilities, even in the absence of possible protein partners, scaffolds, cell debris, or native mammalian membrane bilayers. Future studies of pure protein using other reconstitution systems, such as nanodiscs or liposomes, may be helpful to elucidate the impact of lipids in ligand binding. Liposome systems would also allow us to assess the ability of purified proteins to mediate lipoprotein-cholesterol transport to further validate pure protein function.

Apart from lipoproteins, SR-B1 and CD36 bind a variety of other ligands. For example, both receptors bind to pathogens: CD36 serves as a receptor *Plasmodium falciparum*, a malaria parasite (10), while SR-B1 binds to hepatitis C (50, 51) and has recently been implicated as a possible coreceptor for severe acute respiratory syndrome coronavirus 2 (52). In addition to pathogenic ligands, SR-B1 has also been shown bind serum amyloid A (53), anionic lipids (54), and silica (55) while CD36 binds anionic lipids (54), as well as fatty acids, advanced glycation end products, apoptotic cells, amyloid β, and hexarelin (reviewed in (24)). While not within the scope of this paper, availability of pure protein allows us to use MST assays to probe these expansive binding interactions.

Sf9 cells possess different glycosylation machinery than mammalian cell systems. These differences likely impact the types of sugars added to proteins, as well as their orientations and respective linkages. Both SR-B1 and CD36 expressed at the cell surface in Sf9 cells (Fig. 1), suggesting the glycosylations added by Sf9 cell machinery were sufficient. Previous work has established that glycosylation of SR-B1, specifically at residues N108 and N173, is required for expression at the cell surface (29). Similarly, CD36 requires two groups of asparagine glycosylation sites for expression at the plasma membrane (30). While previous studies utilized mutagenesis strategies to alter single glycosylation sites, our purification system provides the option to remove glycosylations, thus allowing us to investigate the role of this key posttranslational modification in ligand binding. Our MST studies are the first to demonstrate that glycosylation status of SR-B1 or CD36 does not impact binding of lipoprotein ligands. This implicates glycosylation as a processing step required for proper folding or trafficking, but further studies are required to elucidate the precise mechanisms. SR-B1 and CD36 have additional posttranslational modifications, including disulfide bonds, phosphorylation, fatty acylation, and ubiquitination (reviewed in (8, 24)). While some of these modifications have been well-characterized in mammalian cell culture systems, little is known about other modifications. We anticipate the resolution of full-length structures of these receptors will shed insight into the importance of these modifications and how they may impact ligand affinity and lipoprotein-cholesterol transport.

Structural determination of membrane proteins is challenging, but the tools available and number of solved structures are rapidly expanding. The ability to purify full-length SR-B1 and CD36, as well as verify their functionality, creates new opportunities to understand receptor dynamics and provides a necessary step toward understanding the molecular mechanisms behind lipid transport and atherosclerosis development.

## Experimental procedures

Sf9 (*S. frugiperda*) cells were purchased from Expression Systems. Rabbit mAbs targeting the N-terminal extracellular region of SR-B1 (amino acids 50–150) were purchased from Abcam. Rabbit polyclonal antibodies targeting CD36 were purchased from Novus Biologicals. Rat mAbs targeting FLAG were purchased from Agilent. Horseradish peroxidase (HRP)-conjugated donkey-anti-rabbit-immunoglobulin G (IgG) secondary antibody was purchased from GE Healthcare Life Sciences and HRP-conjugated goat-anti-rat-IgG secondary antibody from Santa Cruz Biotechnology. DiI-HDL was obtained from Kalen Biomedical. DiI-oxLDL was purchased from Invitrogen. Human HDL (175–360 kDa) was obtained from MilliporeSigma. To make oxLDL, LDL purchased from Lee Biosciences was oxidized by dialysis against CuSO_4_ in PBS for 6 h at 37 °C, and the reaction was stopped by dialysis in PBS containing 0.54 mM EDTA overnight at 4 °C. Purified apoA-I was a gift from Dr Mary Sorci-Thomas (Medical College of Wisconsin). All other reagents were of analytical grade.

### Plasmid and baculovirus generation

Constructs encoding human full-length SR-B1 (509 amino acids) and human full-length CD36 (472 amino acids) containing a flanking C-terminal hemaglutinin A secretion signal and N-terminal PreScission protease cut site, ten histidine tag, and FLAG tag were synthesized by Twist Bioscience Corporation. pFastBacI dual vector (Thermo Fisher Scientific) was linearized with BamHI and HindIII and human full-length SR-B1 or CD36 constructs were ligated into the pFastBacI vector. pFastBacI plasmids containing SR-B1 or CD36 were transformed into DH10Bac *Escherichia coli* to allow for transposition into bacmid DNA using the Bac-to-Bac Baculoviral Expression System (Invitrogen). Sf9 cells within the logarithmic growth phase were then transfected with bacmid using X-tremeGENE HP DNA Transfection Reagent (MilliporeSigma) to allow for replication of recombinant baculovirus. Recombinant baculoviruses were harvested 5 days post-transfection *via* centrifugation of cultures at 2000*g* at 4 °C for 10 min and the supernatant containing the virus was filtered through a polyethersulfone membrane with a 0.22-μm pore size. Baculovirus was amplified by Sf9 cell infection up to four times to reach high infectious units. Generation and titering of baculoviral stocks were performed as previously described (56).

### Cell culture and infection

Sf9 cells were maintained in suspension in ESF 921 protein-free insect cell culture media (Expression Systems) at 27 °C in room air, shaking at 144 rpm. Cells were split to 1 × 10^6^ cells/ml every 48 h.

### Measurement of DiI-HDL and DiI-oxLDL binding and uptake in Sf9 cells

Sf9 cells (1 × 10^6^ cells/ml) were infected with high-titer baculovirus encoding SR-B1, CD36, or empty vector at a MOI of 5 and immediately plated into 12-well plates. After 72 h, plates for binding were pre-chilled for 10 min at 4 °C. Cells were washed once with 2 ml ESF 921/0.5% bovine serum albumin (BSA) and then incubated for 90 min at 4 °C (to assess binding) or 27 °C (to assess binding and uptake) with 10 μg/ml DiI-HDL or DiI-oxLDL in ESF 921/0.5% BSA. After 90 min, cells were washed in cold PBS and harvested by repeated gentle pipetting in PBS/0.5% BSA. Cells were centrifuged at 300*g* for 3 min and resuspended in 200 μl PBS/0.5% BSA. MFI was recorded on the LSRII (BD Biosciences). DiI uptake was calculated by subtracting the MFI of DiI-HDL or DiI-oxLDL binding at 4 °C from the MFI at 27 °C.

### Cell lysis

Plated Sf9 cells were washed twice in cold PBS (pH 7.4) on ice and lysed in radio-immunoprecipitation assay buffer containing protease inhibitors (1 mg/ml pepstatin, 1 mg/ml leupeptin, 1 mg/ml aprotinin, and 20 mg/ml PMSF) for 10 min on ice. Suspension Sf9 cells were pelleted at 2000*g* for 10 min at 4 °C, the supernatant was removed, and then cells were lysed in radio-immunoprecipitation assay buffer–containing protease inhibitors. Cell lysates were cleared from cellular debris by centrifugation at 8000*g* for 10 min at 4 °C. Lysate protein concentrations were determined by the Lowry method (57).

### Biotinylation of cell surface proteins

Plated Sf9 cells were washed twice in cold PBS (pH 7.4) on ice and incubated with 1 mg/ml EZ-link NHS-Biotin (Thermo Fisher Scientific) in PBS for 1 h at 4 °C. Cells were washed twice in cold PBS and lysed in 1% NP40 buffer containing protease inhibitors for 10 min on ice. Cell lysates were cleared by centrifugation at 8000*g* for 10 min at 4 °C. Protein concentrations of the whole-cell lysate were obtained by the Lowry method (57). Whole-cell lysate (100 μl, approximately ∼100 μg) was mixed with 500 μl 1% NP-40 buffer with protease inhibitors and incubated with 50 μl High Capacity Streptavidin Agarose Resin slurry (Thermo Fisher Scientific) for 1 h at 25 °C. Beads were pelleted by centrifugation at 3500*g* for 2 min and washed five times in 600 μl NP40. Final supernatant was discarded, and pelleted beads were resuspended in 40 μl 2× sample treatment buffer (4% SDS, 20% glycerol, 120 mM β-mercaptoethanol) and 20 μl H_2_O. Samples were boiled for 5 min and 40 μl of each sample was separated by 10% SDS-PAGE.

### Immunoblot analysis

Lysates (15 μg) were combined with an equal volume of Tris–HCl (pH 6.8), 0.005% bromophenol blue, and 10% 2× sample treatment buffer, separated by 10% SDS-PAGE, and wet transferred to a nitrocellulose membrane. Membranes were blocked in 5% milk in tris-buffered saline with Tween (TBST) and probed with anti-SR-B1 (1:1000) antibodies in 1% milk in TBST. For CD36 blots, membranes were blocked in 5% BSA in TBST and probed with anti-CD36 (1:1000) antibodies in 1% BSA or anti-FLAG (1:1000) antibodies in 1% milk in TBST. All membranes were washed and HRP-conjugated donkey-anti-rabbit-IgG secondary antibody (1:10,000) in 1% milk was added for SR-B1 and CD36 blots, while HRP-conjugated goat-anti-rat-IgG secondary antibody (1:10,000) in 1% milk for FLAG blots. Membranes were washed, SuperSignal West Pico Chemiluminescent Substrate (Thermo Fisher Scientific) was added for 5 min, and blots were imaged using a ChemiDoc MP (Bio-Rad).

### Receptor oligomerization *via* PFO-PAGE

Uninfected SR-B1- or CD36-baculovirus infected plated Sf9 cells were washed twice in cold PBS on ice and lysed in PBS containing protease inhibitors for 10 min at 72 h post-baculoviral infection. Cells were sonicated for four 5-s cycles at a power level of 4, and protein concentrations were determined using the Lowry method (57). Lysates (10 μg) were combined with an equal volume of PFO sample treatment buffer (3.2% PFO, 100 mM Tris base, 20% glycerol, 0.005% bromophenol blue), adjusted to pH 8 with NaOH. Standards (100 μg; BSA [66 kDa], alcohol dehydrogenase [150 kDa], catalase [232 kDa], and thyroglobulin [333, 666 kDa]) were combined with sample treatment buffer without PFO. Samples were incubated with PFO sample treatment buffer for 30 min at 25 °C. Lysates and standards were separated by PFO-PAGE on an 8% polyacrylamide gel without SDS and analyzed for SR-B1 or CD36 protein by immunoblot as described above.

### Purification of human full-length SR-B1 and CD36 in Sf9 cells

High-titer baculovirus was used to infect 2 l of Sf9 cells (at a density of approximately 2.5 × 10^6^ cells/ml) at a MOI of 5. Samples were collected in 24 h increments postinfection. At 72 h, 2 l cultures were centrifuged at 2500*g* for 15 min at 4 °C, and pellets were stored at −80 °C until purification. To purify the receptors, thawed pellets were solubilized in 50 ml of low-salt buffer (10 mM Hepes pH 7.5, 10 mM MgCl_2_, 20 mM KCl) and syringe lysed. One cOmplete protease inhibitor tablet (Roche) and iodoacetamide (final concentration of 1 mg/ml) were added and incubated for 30 min at 4 °C. Solubilization buffer (100 ml) (100 mM Hepes, pH 7.5, 0.8 M NaCl, 1.5% LMNG, 0.3% CHS) was added and stirred at 4 °C for 4 h. Lysates were centrifuged at 50,000*g* for 30 min at 4 °C to separate membrane proteins. Supernatant was decanted and 6 ml of TALON resin slurry and imidazole (final concentration of 10 mM) was added. The resin and membrane mixture was nutated overnight at 4 °C, centrifuged at 2000*g* for 10 min at 4 °C, and the supernatant was discarded. Protein-bound resin within the pellet was placed into a polypropylene column and washed five times with 2 ml wash buffer 1 (50 mM Hepes pH 7.5, 400 mM NaCl, 0.1% LMNG, 0.02% CHS, 10% glycerol, 20 mM imidazole) and then washed five times with wash buffer 2 (50 mM Hepes pH 7.5, 400 mM NaCl, 0.025% LMNG, 0.005% CHS, 10% glycerol, 10 mM imidazole). Protein was eluted with 3 ml elution buffer (50 mM Hepes pH 7.5, 400 mM NaCl, 0.025% LMNG, 0.005% CHS, 10% glycerol, 250 mM imidazole). Eluate was concentrated in a 30 kDa MWCO spin concentrator and passed through a desalting column into exchange buffer (50 mM Hepes pH 7.5, 150 mM NaCl, 0.025% LMNG, 0.005% CHS), removing excess imidazole. To deglycosylate proteins, 25 μl of PNGase F was added to the desalting column elution and the mixture was nutated overnight at 4 °C or for 2 h at 37 ˚C. To remove excess PNGase F and concentrate the final protein product, samples were concentrated in a 50 kDa MWCO spin concentrator. Protein concentrations were calculated from absorbance readings at 280 nm using a Nanodrop spectrophotometer.

### Size-exclusion chromatography

Purified protein (CD36 or SR-B1) was dialyzed into 0.02 μm filtered exchange buffer overnight. An SEC column (BioSep-SEC-S 4000, 300 × 7.8 mm) fitted with a guard column (08543-TSKgel Guard SWXL, 6 mm ID × 4 cm, 7 μM) was equilibrated with 0.02 μm filtered exchange buffer overnight. Protein (100 μl, 0.20 mg/ml) was injected onto the column with a flow rate of 1 ml/min at 25 °C for 30 min. Eluant was monitored by measuring absorbance at 220 and 280 nm.

### Thermal shift assay

Purified SR-B1 and CD36 stability in exchange buffer was measured weekly for 6 weeks postpurification by Prometheus NT.48. Ten microliters of receptor at a concentration of approximately 1 mg/ml was loaded into Prometheus standard capillaries. Protein unfolding was monitored by assessing changes in intrinsic tryptophan fluorescence at 330 and 350 nm over a temperature gradient (0.5 °C/min) from 20 °C to 90 °C at 20% power. The destabilization temperatures were calculated by taking the ratio of the first derivative values of the sample fluorescence at 350 and 330 nm as described (58, 59, 60).

### Microscale thermophoresis

Purified SR-B1 or CD36 protein was labeled using the RED-NHS 2nd Generation Protein Labeling Kit (NanoTemper Technologies) according to manufacturer’s instructions. Samples were prepared in exchange buffer and loaded into standard capillaries. Measurements were performed on the Monolith NT.115 BLUE/RED (NanoTemper Technologies) at 25 °C using 40% MST power and laser on/off times of 0 s and 21 s, respectively. Labeled receptor (SR-B1 or CD36) was diluted in exchange buffer to a concentration of 20 nM. For each ligand, 10 μl of starting concentration ligand was diluted 1:1 in 10 μl of exchange buffer to make a 16-sample dilution series. Ten microliters of receptor at constant concentration (20 nM) was incubated with 10 μl of ligand at each dilution in the series, briefly centrifuged, and loaded into Monolith NT.115 capillaries. Each sample was checked for sample aggregation and capillary adsorption and points with either were removed. All measurements were performed with protein from two separate protein purifications. The MST technique is described in further detail (27). Apparent K_d_ values were calculated by nonlinear regression analysis, assuming one-site specific binding, of the normalized thermophoresis (∂Fnorm), using GraphPad Prism (https://www.graphpad.com/).

### Data presentation, analysis, and statistics

All graphs were generated using GraphPad Prism 9.4. Bar graphs are represented as mean ± SD, with each individual values plotted. Means were compared by one-way ANOVA and Dunnett’s multiple comparisons test.

## Data availability

All data are contained in the manuscript.

## Conflicts of interest

B. F. V. has ownership interests in Protein Foundry, LLC and XLock Biosciences, LLC. The other authors declare that they have no conflicts of interest with the contents of this article.
